# Proconvulsant effects of the ketogenic diet in electroshock-induced seizures in mice

**DOI:** 10.1007/s11011-016-9900-4

**Published:** 2016-09-20

**Authors:** Iwona Zarnowska, Jarogniew J. Luszczki, Tomasz Zarnowski, Piotr Wlaz, Stanislaw J. Czuczwar, Maciej Gasior

**Affiliations:** 1 Department of Pathophysiology, Medical University, Jaczewskiego 8, 20-090 Lublin, Poland; 2 Department of Physiopathology, Institute of Agricultural Medicine, Jaczewskiego 2, 20-950 Lublin, Poland; 3 Chair of Ophthalmology, Medical University, Chmielna 1, 20-079 Lublin, Poland; 4 Department of Animal Physiology, Institute of Biology and Biochemisry, Faculty of Biology and Biotechnology, Maria Curie-Skłodowska University, Akademicka 19, 20-033 Lublin, Poland; 5 Department of Pharmacology and Physiology, Drexel University College of Medicine, Philadelphia, PA USA

**Keywords:** Ketogenic diet, Epilepsy, Antiepileptic drugs, Protection

## Abstract

Among non-pharmacological treatments, the ketogenic diet (KD) has the strongest demonstrated evidence of clinical success in drug resistant epilepsy. In an attempt to model the anticonvulsant effects of the KD pre-clinically, the present study assessed the effects of the KD against electroshock-induced convulsions in mice. After confirming that exposure to the KD for 2 weeks resulted in stable ketosis and hypoglycemia, mice were exposed to electroshocks of various intensities to establish general seizure susceptibility. When compared to mice fed the standard rodent chow diet (SRCD), we found that mice fed the KD were more sensitive to electroconvulsions as reflected by a significant decrease in seizure threshold (3.86 mA in mice on the KD vs 7.29 mA in mice on the SRCD; *P* < 0.05) in the maximal electroshock seizure threshold (MEST) test. To examine if this increased seizure sensitivity to electroconvulsions produced by the KD would affect anticonvulsant effects of antiepileptic drugs (AEDs), anticonvulsant potencies of carbamazepine (CBZ), phenobarbital (PB), phenytoin (PHT), and valproate (VPA) against maximal electroshock (MES)-induced convulsions were compared in mice fed the KD and SRCD. We found that potencies of all AEDs studied were decreased in mice fed the KD in comparison to those on the SRCD, with decreases in the anticonvulsant potencies ranging from 1.4 fold (PB) to 1.7 fold (PHT). Finally, the lack of differences in brain exposures of the AEDs studied in mice fed the KD and SRCD ruled out a pharmacokinetic nature of the observed findings. Taken together, exposure to the KD in the present study had an overall pro-convulsant effect. Since electroconvulsions require large metabolic reserves to support their rapid spread throughout the brain and consequent generalized tonic-clonic convulsions, this effect may be explained by a high energy state produced by the KD in regards to increased energy storage and utilization.

## Introduction

Drug resistant epilepsy continues to be a significant unmet medical need despite the availability of more than 25 approved antiepileptic drugs (AEDs) (Loscher et al. 2013). In addition to a continuous effort to improve current AEDs and discover new small molecules (Bialer et al. 2015), non-pharmacological treatment approaches are increasingly being considered for the treatment of drug refractory epilepsy (Engel 2014; Sharma et al. 2015). Among those, the dietary approach by substituting the regular diet with the ketogenic diets (KDs) has the strongest evidence so far of clinical success in drug resistant epilepsy (Li et al. 2013; Reid et al. 2014). This has fueled interest in understanding mechanisms responsible for KD’s anticonvulsant and perhaps disease-modifying (i.e., antiepileptogenic) properties (Allen et al. 2014; Danial et al. 2013; Gasior et al. 2006; Hartman et al. 2007; Masino and Rho 2012). One reductional way of studying the KD pre-clinically is by evaluating its anticonvulsant profile across many animal models to look for a unique phenotypic signature that would differentiate the KD from clinically approved AEDs.

Animal models of seizures and epilepsy syndromes have been pivotal in pre-clinical testing of treatments of epilepsy since the Merritt and Putnam’s demonstration in 1937 of the anticonvulsant efficacy of phenytoin in a cat model of seizures induced by an electric shock; since then no AED has been approved without pre-clinical evidence of its anti-seizure efficacy (Smith et al. 2007; Gasior and Wiegand 2012; Bialer et al. 2015). Although there are many seizure tests and epilepsy models available, the initial assessment of anticonvulsant effects of a treatment often begins with tests that use an acute electrical stimulation (e.g. maximal electroshock, MES, test or closely related the maximal electroshock seizure threshold, MEST, test) or a chemical agent (e.g. pentylenetetrazol, PTZ) to induce seizures in mice or rats (Giardina and Gasior 2009; Smith et al. 2007). Not surprisingly, the MES and MEST tests had also been used in early (Appleton and De Vivo 1973; Appleton and DeVivo 1974; Davenport and Davenport 1948; Millichap et al. 1964; Nakazawa et al. 1983; Otani et al. 1984; Uhlemann and Neims 1972) and more recent (Bough and Eagles 2001; Bough et al. 2000; Likhodii et al. 2000; Thavendiranathan et al. 2000, 2003) studies on the anticonvulsant properties of the KDs. Unfortunately, those studies revealed mixed results (i.e. ranging from no effect to anticonvulsant or proconvulsant) on the effects of the KDs on seizures in the MES or MEST seizure tests, which is the case when tested and compared across other experimental settings (Hartman et al. 2007; Samala et al. 2008; Masino and Rho 2012).

The aim of the present study was to assess the effects of the classic KD most commonly used in clinics (i.e. based on long-chain fatty acids) against the electroshock-induced convulsions in mice. Given the contradictory data on the effects of the KD on electroshock-induced convulsions from the literature, no a priori hypothesis was set to be verified. Specifically, effects of the KD were first assessed in the MEST seizure test that allowed detection of both pro- and anticonvulsant properties of a tested treatment modality (Giardina and Gasior 2009). Once pro-convulsant effects of the KD in the MEST seizure test were established, we sought to establish how it would affect the anticonvulsant effects of several AEDs in the MES seizure test. Here, we report for the first time that the KD weakens the acute anticonvulsant effects of carbamazepine, phenobarbital, phenytoin, and valproic acid in the MES seizure test without altering their brain exposures in mice. This paradoxical pharmacodynamic effect of the KD is discussed in the context of using the MES seizure test for evaluating anticonvulsant effects of the KD.

## Materials and methods

### Animals

Male Swiss mice weighing 20–25 g were kept in an environmentally-controlled vivarium (temperature and relative humidity, 21 ± 1 °C and 55 ± 3 %, respectively) operating under a natural light-dark cycle. The animals were housed in colony cages with free access to food (chow pellets) and tap water. Only experimentally naive mice were used and experimental groups consisted of at least 8 mice per group. All tests were performed between 9.00 a.m. and 2.00 p.m. Procedures involving animals and their care were conducted in accordance with the European Communities Council Directive of September 22, 2010 (2010/63/EU) and Polish legislation on animal use in biomedical experiments. The experimental protocols and procedures listed below were approved by the First Local Ethics Committee in Lublin and conformed to the *Guide for the Care and Use of Laboratory Animals (* *www.nap.edu/readingroom/books/labrats* *)*.

### Ketogenic diet (KD)

All mice were acclimated to the facility on the regular rodent chow diet for at least 7 days. Then, all mice were fasted overnight before being randomly assigned to groups fed either a KD or continued on the standard rodent chow diet (SRCD) for the next 2 weeks. The KD used in the present study (Bio-Serv F3666; 8.6:1 ratio of fat to proteins + carbohydrates) has been described elsewhere (Bough and Eagles 1999; Bough et al. 2000; Hartman et al. 2008).

Weights of the animals maintained on each diet were monitored throughout the study. In addition, KD-induced ketosis and glucose levels were monitored. Those measures were performed in mice that were not treated with any drugs nor underwent any seizure testing. Trunk blood glucose and β-hydroxybutyrate levels were measured with a Precision Xtra Advanced Diabetes Management System with Precision Xtra test strips for measuring blood glucose and β-hydroxybutyrate levels (Abbott Diabetes Care Inc., Alameda, CA) as described by (Hartman et al. 2008; Schwechter et al. 2003). Monitors were calibrated at the start of each experiment. Before collecting blood samples, animals were euthanized in a CO_2_-infused plastic chamber. Plasma concentrations of glucose and β-hydroxybutyrate were expressed in mg/ml and mmol/ml, respectively.

### Drugs

The following AEDs were used: carbamazepine (CBZ; a gift from Polfa, Starogard Gdanski, Poland), phenobarbital (PB; Polfa, Krakow, Poland), phenytoin (PHT; Polfa, Warszawa, Poland), and valproic acid (VPA; as magnesium salt; kindly donated by ICN Polfa, Rzeszow, Poland). CBZ, PB, and PHT were suspended in a 1 % aqueous solution of Tween 80 (Sigma, St. Louis, MO, USA) in sterile saline; VPA was dissolved in sterile saline. Doses of AEDs were expressed as mg/kg body weight. The AEDs were administered as follows: PHT at 120 min, PB at 60 min, CBZ and VPA - 30 min before seizure testing and sampling for pharmacokinetic studies. The selected pretreatment times for the AEDs correspond to their peak anticonvulsant activity based on the available literature and previous experiments.

### Electroconvulsions

Electroconvulsions were induced by applying an alternating current (50 Hz; maximum output voltage 500 V) via ear-clip electrodes from a rodent shocker generator (type 221; Hugo Sachs Elektronik, Freiburg, Germany). The stimulus duration was 0.2 s. Tonic hindlimb extension (i.e., rigid extension of the hindlimbs that exceeds a 90° angle with the body) was taken as the endpoint. This apparatus was used to induce seizures in two methodologically different experimental approaches: maximal electroshock seizure threshold (MEST) test and maximal electroshock seizure (MES) test (Giardina and Gasior 2009; Zarnowska et al. 2009).

### MEST test

Effects of the KD on the threshold for electroconvulsions were first assessed in the MEST test. Specifically, separate groups of mice (*n* ≥ 8 mice per group) were exposed to currents of varying intensities until data were collected with at least three current intensities at which close to 0, 50, and 100 % of animals exhibited the endpoint. After establishing the current intensity-effect curve (i.e., current intensity in mA vs. percentage of mice convulsing) in mice maintained on the KD or regular diet, the electroconvulsive threshold was calculated according to a log-probit method by (Litchfield and Wilcoxon 1949). The electroconvulsive threshold was expressed as the median current strength value (CS_50_) in mA predicted to produce tonic hindlimb extension in 50 % of the animals tested (Giardina and Gasior 2009).

### MES test

In the MES test, mice were challenged with a current of the fixed intensity (25 mA) that was 4-5-fold higher than the CS_50_ value in vehicle-treated control mice (Löscher et al. 1991). These parameters of stimulation (so called maximal electroshock) typically result in all mice to respond with tonus immediately after stimulation. The AEDs administered in mice fed either the control diet or KD were tested for their ability to increase the number of animals not responding with tonus (i.e., protected from tonus) after stimulation. Again, at least three groups of mice, each consisting of at least 8 animals and treated with a different dose of the AED alone, were challenged to collect data where close to 0, 50, and 100 % of animals were protected from tonic seizures. After constructing a dose-effect curve (i.e., dose in mg/kg vs. percentage of mice protected), the protective median effective dose (ED_50_) value of the drug tested was calculated according to a log-probit method (Litchfield and Wilcoxon 1949). Each ED_50_ value represented a dose of the test drug (in mg/kg) predicted to protect 50 % of mice tested against MES-induced extension of the hindlimbs. In this experimental protocol, an increase in the anticonvulsant potency of the AED tested would be reflected by a lower ED_50_ value of that AED (i.e., its lower dose necessary to protect 50 % of mice challenged). Conversely, a decrease in the anticonvulsant potency of the AED tested would be reflected by a higher ED_50_ value of that AED (i.e., its higher dose necessary to protect 50 % of mice challenged).

### Measurement of total brain AED concentrations

Total brain concentrations of the selected AEDs were measured at doses corresponding to their ED_50_ or the highest dose tested in the MES test (*n* = 8 mice per group). Specifically, mice pretreated with a given AED from groups of mice exposed to the SRCD or KD were decapitated and the whole brain was collected, weighed, and homogenized using Abbott buffer (1:2 weight/volume) in an Ultra-Turrax T8 homogenizer (IKA-Werke, Staufen, Germany). The homogenates were then centrifuged at 10,000*g* for 10 min and the supernatant samples of 100 μl were collected and analyzed for AED content. Total brain concentrations of the classical AEDs (CBZ, PB, PHT, and VPA) were measured by a fluorescence polarization immunoassay (FPIA) using an analyzer (Abbott TDx) and manufacturer-supplied reagent kits (Abbott Laboratories, North Chicago, IL, USA). At least 8 animals were used per each treatment group and brain concentrations of AEDs were expressed as group means ± S.D. (*n* = 8) in μg/ml of brain supernatant. Plasma levels of glucose and β-hydroxybutyrate were expressed as group means (± SEM). The CS_50_ values in the MEST test and the protective ED_50_ values of AEDs in the MES test were calculated using the log-probit method by (Litchfield and Wilcoxon 1949), followed by the method transforming 95 % confidence limits to standard error of the mean (Luszczki et al. 2006). Statistical analysis of data from the MEST and MES tests was performed with one-way ANOVA followed by the Tukey-Kramer test for multiple comparisons. Total brain concentrations of AEDs were statistically analyzed using the unpaired Student’s *t*-test or one-way ANOVA followed by the post-hoc Bonferroni’s test for multiple comparisons. Statistical tests were performed using GraphPad Prism version 4.0 (GraphPad Software, San Diego, CA, USA). Differences were considered statistically significant at *P* < 0.05.

## Results

### Effect of the KD on the overall behavior and biochemical parameters

Exposure to the KD for 2 weeks did not have any apparent deleterious effect on mice behavior except for some (approximately 25–30 %) weight loss that was comparable to the weight loss reported in Hartman et al. 2008. Development of ketosis and hypoglycemia (as measured by plasma levels of β-hydroxybutyrate and glucose, respectively) in mice fed the KD for 2 weeks were confirmed (β-hydroxybutyrate: 2.3 ± 0.3 mM, *N* = 23; glucose: 68.7 ± 5.4 mg/ml; *N* = 16).

### Effect of the KD on the threshold for electroconvulsions in the MEST test

Mice maintained on the KD for 2 weeks were more sensitive to electroconvulsions as reflected by a significant decrease in seizure threshold in the MEST test (CS_50_ in mice fed the KD versus regular diet: 3.86 mA (95 % confidence limits: 3.24 – 4.60; *N* = 24) versus 7.29 mA (95 % confidence limits: 5.25 – 10.14; *N* = 24); *P* < 0.05 (Litchfield and Wilcoxon 1949)).

### Effect of the KD on the anticonvulsant action of AEDs in the MES test

**Fig. 1:**
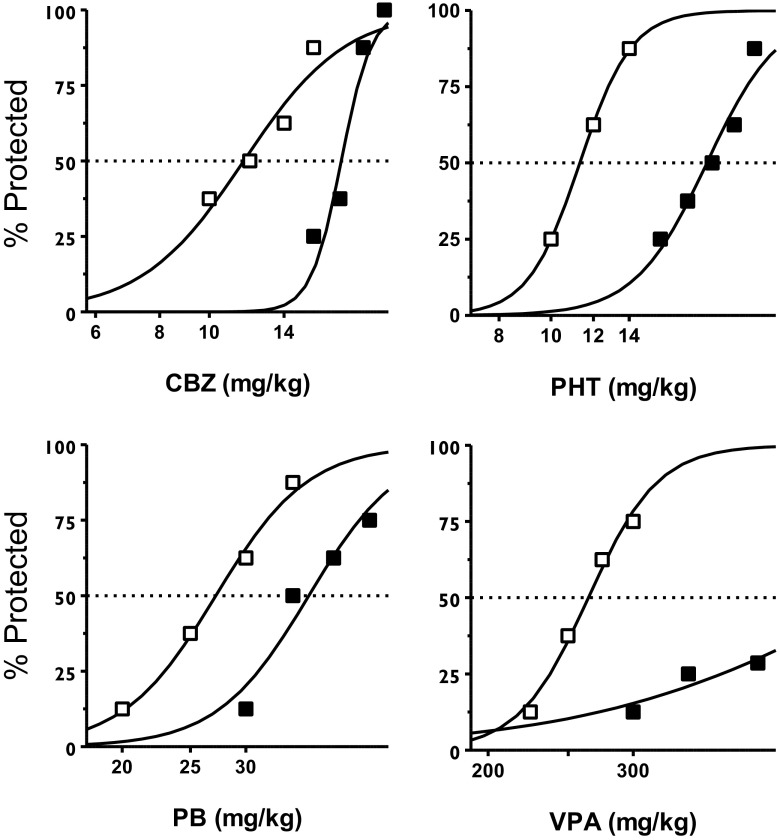
Dose-effect function of CBZ, PB, PHT, and VPA in mice maintained on either the regular diet (□) or ketogenic diet (■) against MES-induced seizures in mice. Each data point represents percent of mice protected (*N* = at least 8 mice/data point) at a given dose (doses in mg/kg on abscissa). Sigmoidal curves are the result of a least squares fit of dose-response function for each AED under each diet regimen. Points of intersections with the *dashed line* at 50 % correspond to approximate ED_50_ values of AEDs; note however that the log-probit method was used for calculating ED_50_ presented in Table 1 (Litchfield and Wilcoxon 1949); see Table 1 for the calculated ED_50_ values for each AEDs and other details

The anticonvulsant dose-effect function and resulting anticonvulsant ED_50_ values for each AEDs tested in the MES test are showed in Fig. 1 and Table 1, respectively. The anticonvulsant effects of all AEDs studied were diminished in mice fed the KD in comparison to those maintained on the regular diet. This effect was reflected by right-ward shifts of the anticonvulsant dose-effect functions for CBZ, PB, PHT, and VPA in mice fed the KD in comparison to mice fed the regular diet (Fig. 1); these resulted in significant decreases in the anticonvulsant potencies as measured by ED_50_ values (Table 1). Decreases in the anticonvulsant potencies ranged from 1.4 fold (PB) to 1.7 fold (PHT). In the case of VPA, its anticonvulsant ED_50_ value in mice fed the KD could not be established due to dose-limiting toxicity of VPA at doses higher than 400 mg/kg. Of note, only 25 % of mice were protected at the dose 400 mg/kg of VPA when fed the KD, whereas 50 % of mice fed the regular diet were protected by an estimated dose of VPA 265.9 mg/kg; this would approximate at least a 1.5-fold shift (i.e., increase) in VPA’s ED_50_ value in mice fed the KD in comparison to those fed the regular diet.

**Table 1:** Effect of the ketogenic diet (KD) on the protective activity of carbamazepine (CBZ), phenytoin (PHT), phenobarbital (PB) and valproate (VPA) against maximal electroshock-induced seizures in mice

Diet	AED	PT (min)	AED ED_50_ (mg/kg)	Potency ratio
SRCD	CBZ	30	11.4 (9.6–13.6)	1.56 (1.29–1.88)
KD	CBZ	30	17.7 (16.5–19.1)*	
SRCD	PB	60	26.9 (22.7–31.9)	1.39 (1.13–1.70)
KD	PB		37.4 (33.5–41.8)*	
SRCD	PHT	120	11.3 (10.0–12.9)	1.71 (1.45–2.01)
KD	PHT	120	19.4 (17.4–21.5)*	
SRCD	VPA	30	265.9 (243.5–290.3)	≥1.5
KD	VPA	30	≥400	

Results are presented as median effective doses (ED_50_ in mg/kg; with 95 % confidence limits in parentheses) required to protect 50 % of animals tested against maximal electroshock-induced seizures and potency ratios (with 95 % confidence limits) calculated by dividing AED’s ED_50_ value in mice fed the KD by AED’s ED_50_ value in mice fed the SRCD. All calculations and statistical comparisons were made by a log-probit method (Litchfield and Wilcoxon 1949). ED_50_ of VPA could not be established due to dose-limiting toxicity. Animals were maintained on ketogenic diet (KD) for at least 14 days. Each antiepileptic drug (AED) was administered i.p. at the pre-selected time before seizure testing (second column)

*CBZ* carbamazepine, *PB* phenobarbital, *PHT* phenytoin, *VPA* valproic acid, *KD* ketogenic diet

*P < 0.05 vs. mice fed the SRCD

### Effects of the KD on brain levels of AEDs

Exposure to the KD for 2 weeks had no effect on the brain levels of the four AEDs studied (Table 2).

**Table 2:** Effect of the KD administered for 14 days on total brain AED concentrations

Diet	AED ED_50_ (mg/kg)	Brain concentration (μg/ml)	*P* value
SRCD	CBZ (17.7)	4.24 ± 0.81	>0.05
KD	CBZ (17.7)	4.27 ± 0.79	
SRCD	PB (37.4)	15.01 ± 2.05	>0.05
KD	PB (37.4)	14.70 ± 1.99	
SRCD	PHT (19.4)	2.63 ± 0.65	>0.05
KD	PHT (19.4)	2.40 ± 0.78	
SRCD	VPA (400)	120.6 ± 11.0	>0.05
KD	VPA (400)	119.7 ± 15.9	

Data are presented as means ± S.D. (*N* = 8) in μg/ml of brain supernatant. Statistical evaluation of data was performed with unpaired Student’s *t*-test. For more details see Table 2

## Discussion

In the present study, mice fed the KD for 2 weeks developed ketosis and hypoglycemia comparable to earlier reports in which KD’s anticonvulsant effects were demonstrated (Hartman et al. 2008). Mice fed the KD for 2 weeks were more susceptible to seizures induced in the MEST seizure test. Also, anticonvulsant potencies of CBZ, PB, PHT, and VPA were decreased in the MES seizure test in mice fed the KD. Effects of the KD on potencies of AEDs studied were not due to pharmacokinetic interactions since the KD did not change AEDs’ bioavailability in the brain at the times of their testing in the MES seizure test. That the KD similarly affected all AEDs studied with both overlapping and distinct mechanisms of action (Bialer et al. 2015) suggests that the observed enhanced seizure propensity to electroconvulsions induced by the KD was not due any particular mechanism affected; instead it was more likely representing a non-specific change in the brain. Taken together, exposure to the KD in the present study had an overall pro-convulsant effect when tested in mice when seizures were acutely induced by an electric shock.

Clinical evidence supporting efficacy of the KD in refractory epilepsy is undeniable (Li et al. 2013; Giordano et al. 2014; Reid et al. 2014). However, establishing efficacy of the KD under experimental conditions in animals has repeatedly proven to be a challenge (Hartman et al. 2007). The MEST and MES seizure tests were used before in mice and rats exposed to different KDs and the results of those studies provided only an inconsistent pattern of responses to this convulsive stimulus with nearly as many reports showing no efficacy of the KD against electroconvulsions as there are reports showing its anticonvulsant or pro-convulsant effects (see Introduction). Reconciling reasons for those discrepant results would be difficult now due to different experimental procures used in those reports (e.g. different species or strains within the same species, age of animals, KD compositions, durations to KD of exposure, or parameters of electroshock seizure stimulation).

The core of changes produced by the KDs includes ketosis, hypoglycemia, and an increased overall metabolic state of neurons. It is well documented that ketone bodies, and acetone in particular, have anticonvulsant properties across many seizure tests including those induced by electroconvulsions (Gasior et al. 2008; Likhodii et al. 2003; Rho et al. 2002). Thus, ketosis is unlikely to be the cause of increased seizure sensitivity to electroconvulsions as observed in the present study. On the contrary, ketosis would rather be more likely to offer some attenuation of the KD-facilitated spread of seizures induced by an electric shock.

The role of low glucose levels in seizure sensitivity is more complex (Schwechter et al. 2003). Low glucose levels are necessary for maintaining seizure suppression in animals and patients on the KD (Greene et al. 2001; Huttenlocher 1976). On the other hand, low blood glucose levels as induced by insulin excess, for example, can result in seizures in humans (Cryer 1999; Malouf and Brust 1985). Similarly, low glucose precipitates seizure activity or lowers seizure threshold in animals (Kaul et al. 1980; Kirchner et al. 2006; Reid et al. 2011; Waltregny et al. 1966). Of note, 2-Deoxy-D-glucose, a glucose analog that accumulates in cells and interferes with carbohydrate metabolism by inhibiting glycolytic enzymes, promotes seizures in the MEST seizure test while behaving as an anticonvulsant in other seizure tests (Gasior et al. 2010; Sutula et al. 2006). Taken together, these findings point to the role of low glucose levels in increasing seizure susceptibility, and that the seizure tests utilizing electroconvulsions may be particularly useful for revealing this effect.

KD diets have been shown to increase energy storage and its utilization efficiency (Bough 2008; Bough et al. 2006). Electroshock is a way of applying an electric stimulation to the brain. Electrical simulation with high current intensities such as those used in the MEST and MES tests results in almost instantaneous activation of the brainstem followed by a rapid spread of neuronal activation to other brain regions and to the spinal cord to finally manifest as observable convulsions (Browning et al. 1981a, b; Eells et al. 2004; Peterson 1998). After the electroshock is delivered, seizure activity in the brain and consequent convulsions are induced within milliseconds to seconds. This rapid electrophysiological response is likely to be possible only when neurons and their connections are physiologically viable, as is the case when healthy, non-epileptic animals are used in seizure testing (Gasior and Wiegand 2012). Second, this excessive neuronal activity is likely to rapidly increase the rate of energy use and thus must rely on sufficient energy reserves to support itself (Fujikawa et al. 1989; Meldrum and Chapman 1999; Wasterlain et al. 2010; Yang et al. 2013). Indeed, KDs, due to their effect on increasing energy storage and utilization efficiency, may inadvertently create an environment that facilitates spread of high energy-demanding electroconvulsions, as seen in the present study.

In summary, the results of the present study show that mice fed the KD become more sensitive to electroconvulsions under experimental parameters such as those used in this study. Since electroconvulsions require large metabolic reserves, this effect may be explained by a high energy state produced by the KD in regards to increased energy storage and utilization. Demonstration of the pro-convulsant effects of the KD against seizures induced by electroconvulsions does not diminish the utility of MES/MEST-based seizure tests in pre-clinical testing of potential anticonvulsant therapies. On the contrary, one can argue that these seizure tests may experimentally differentiate therapies that work through targets classically linked to epilepsy (eg, GABA, NMDA/AMPA, Na^+^, Ca^2+^, K^+^) from those that affect, for example, neuronal metabolism. Lastly, the results reported herein have an effect beyond epilepsy in that they provide a pre-clinical warning signal for the use of the electroconvulsive therapy in psychiatric patients who are on the KD seeking its potential therapeutic benefit or as a lifestyle choice (e.g. for weight control).

## Abbreviations

AEDs: Antiepileptic drugs
CBZ: Carbamazepine
KD: Ketogenic diet
MES: Maximal electroshock
MEST: Maximal electroshock seizure threshold
PB: Phenobarbital
PHT: Phenytoin
VPA: Valproate
